# Rasch-Built Overall Amyotrophic Lateral Sclerosis Disability Scale as a Novel Tool to Measure Disease Progression

**DOI:** 10.3390/biomedicines13010178

**Published:** 2025-01-13

**Authors:** Can Sun, Yong Chen, Lu Xu, Wenjing Wang, Nan Zhang, Christina N. Fournier, Nan Li, Dongsheng Fan

**Affiliations:** 1 Department of Neurology, Peking University Third Hospital, Beijing 100191, China; sc@bjmu.edu.cn (C.S.); ronaldchen@bjmu.edu.cn (Y.C.); jingwang@bjmu.edu.cn (W.W.); 13520985887@163.com (N.Z.); 2 Beijing Municipal Key Laboratory of Biomarker and Translational Research in Neurodegenerative Diseases, Beijing 100191, China; 3 Key Laboratory for Neuroscience, National Health Commission/Ministry of Education, Peking University, Beijing 100191, China; 4 Research Center of Clinical Epidemiology, Peking University Third Hospital, Beijing 100191, China; luxu@bjmu.edu.cn; 5 Department of Neurology, Emory University, Atlanta, GA 30322, USA; christina.nicole.fournier@emory.edu

**Keywords:** amyotrophic lateral sclerosis, scale, disease progression, outcome measure

## Abstract

**Background:** A valuable outcome measure to monitor amyotrophic lateral sclerosis (ALS) disease progression is crucial in clinical trials. Rasch-Built Overall Amyotrophic Lateral Sclerosis Disability Scale (ROADS) is a novel questionnaire assessing ALS disability. Currently, there are no studies on the relationship between ROADS and ALS survival. This study explored the value of Chinese ROADS as a novel tool for measuring disease progression and the correlation between ROADS and ALS survival. **Methods:** A total of 170 ALS participants were included in this study. Clinical characteristics and baseline ROADS, ΔROADS, ALSFRS-R, and ΔFRS of patients were collected. Participants were followed for 18 months to assess time to tracheostomy and survival. Scales were collected every 3 to 6 months. We evaluated the association of baseline ROADS and ΔROADS with survival using Cox regression analyses. Linear mixed effects models were used to assess changes over time in ROADS and ALSFRS-R. **Results:** Multivariate Cox models confirmed that baseline ROADS positively correlated with ALS survival (HR = 0.95, *p* < 0.001), while baseline ΔROADS negatively correlated with survival (HR = 1.26, *p* < 0.001). Additionally, linear mixed effects models suggested that ROADS, similar to ALSFRS-R, declined significantly over time, but there was no significant difference between these two. **Conclusions:** Our study indicates that Chinese ROADS is strongly related to ALS survival. Changes in ROADS with disease progression are similar to those in ALSFRS-R. These findings support Chinese ROADS as a reliable outcome measure for clinical trials, potentially enhancing the dimension of evaluating treatment effectiveness in ALS trials.

## 1. Introduction

Amyotrophic lateral sclerosis (ALS) is a highly heterogeneous and rapidly progressive disease with limited treatment options [1]. A major obstacle to evaluating new treatments is the lack of sensitive outcome measures and specific biomarkers to monitor disease progression. The Amyotrophic Lateral Sclerosis Functional Rating Scale-revised (ALSFRS-R) is the most commonly used tool for assessing ALS severity and progression in clinical settings [2]. It is also frequently used as a primary outcome measure in ALS clinical trials [3,4,5,6,7]. The ALSFRS-R and its rate of change (ΔFRS) have proved to be independent predictors of survival for ALS patients [8,9,10]. However, a bulk of evidence has shown problems with ALSFRS-R. Limitations of ALSFRS-R include multidimensionality [11,12], meaning the scale is measuring other factors besides functional status, non-linearity [13], meaning that a one-point change can represent a small or a large change in function depending on the item, and lack of scale responsiveness [14,15], meaning a scale’s ability to capture change when clinical change has actually occurred. Therefore, the quest for reliable measures to monitor disease progression and predict outcomes remains a critical endeavor [16].

Based on the above background, Fournier et al. developed and validated a new scale by Rasch analysis in 2020, assessing overall disability for ALS patients, called the Rasch-Built Overall Amyotrophic Lateral Sclerosis Disability Scale (ROADS) [17]. Rasch methodology, based on modern test theory techniques, is used to develop new scales with advantages over conventional ordinal scales developed by classic theory approaches [18,19,20,21,22]. ROADS is a unidimensional, linear-weighted scale and has improved item targeting compared with ALSFRS-R [17]. It is a 28-question self-reported questionnaire with each item scored 0, 1, or 2. We previously translated ROADS into a Chinese version and validated it by Rasch analysis [23]. More recently, it has been translated into Italian [24] and Spanish [25] versions. The ROADS exhibits promising potential for clinical application. However, longitudinal studies on ROADS have been limited to date [26,27,28]. Notably, the capability of the Chinese ROADS version to measure disease progression and assess prognosis remains unverified. Our study employed a prospective, observational design to evaluate the performance of the Chinese ROADS in monitoring disease progression and to assess its correlation with ALS survival.

## 2. Materials and Methods

### 2.1. Participants and Procedures

**Figure 1:**
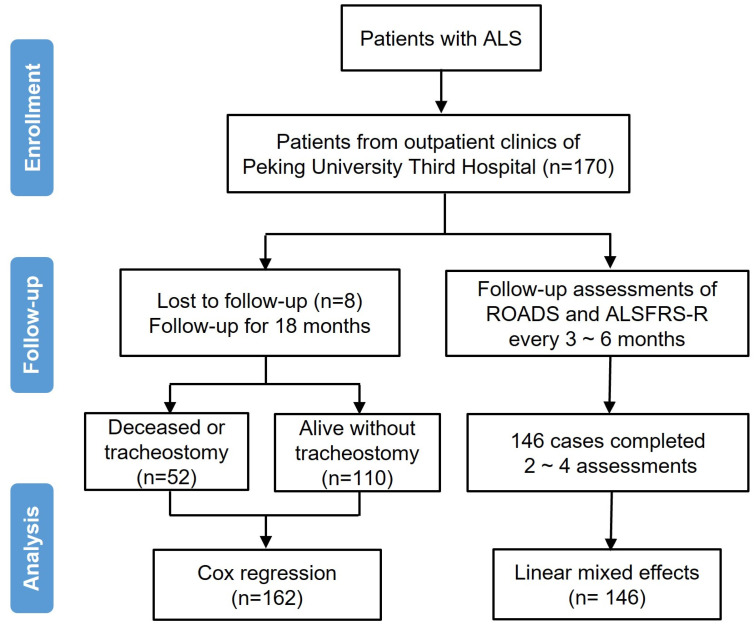
Study procedures and flow diagram in the study.

Patients diagnosed with ALS, according to the revised EI Escorial criteria [29], were enrolled into this study from our previous research [23] at Peking University Third Hospital, Beijing, China. The follow-up period lasted approximately 18 months, from February 2020 to 6 September 2021 [25]. We collected baseline data including age, gender, site of onset, symptom duration, ALSFRS-R, ROADS, ΔFRS, and ΔROADS. ΔFRS was calculated according to a previous study [9]. We defined ΔROADS with the reference of ΔFRS. ΔROADS = (141 − (Total Normed ROADS at baseline))/(Symptom duration (months)). Symptom duration was defined as the time from reported onset to the baseline visit. The survival endpoint was identified as tracheostomy or death. Survival time was the time from the baseline to the endpoint. To validate the ability of ROADS to change with disease progression, we conducted three follow-up assessments of two scales during the 18-month period after collecting the baseline data. The ALSFRS-R was administered by trained neurologists and the ROADS was filled out by themselves. Figure 1 summarized the study flow. This study has been approved by the Research Ethics Committee of Peking University Third Hospital. All participants provided written informed consents.

### 2.2. Statistical Analyses

The ROADS values were recorded as normed scores converted from logit units in the original article, which were linearly weighted [17,23]. Baseline demographic and clinical characteristics of those who reached the endpoint and those who did not were compared. For continuous variables, we performed the Student’s *t*-test for normally distributed data and the Wilcoxon’s rank sum test for non-normal data. Normality was confirmed by performing Shapiro–Wilk test. Chi-square test was conducted for categorical variables. Associations of ROADS and ΔROADS score at the baseline with the survival time were assessed using Cox regression. Univariate Cox regressions followed by multivariate Cox regressions were performed to determine independent prognostic factors. Baseline variables that were considered clinically relevant or that showed a univariate relationship with the outcome (*p* < 0.2) were entered into multivariate Cox regression models, including age, gender, site of onset, and symptom duration. Adjusted hazard ratios (HRs) with 95% confidence intervals (CIs) were calculated. Schoenfield residual analysis was used to assess the proportional hazard assumption. Multicollinearity was assessed with the variance inflation factor (VIF). First, ROADS, ΔRAODS, ALSFRS-R, and ΔFRS were included in the Cox models as continuous variables. Then, we transformed them into categorical variables based on tertiles and incorporated them into Cox models.

Longitudinal rates of decline in ROADS were analyzed by linear mixed effects models. To allow direct comparisons between ALSFRS-R and ROADS, the two scores were Z-score normalized by subtracting the mean and dividing by the standard deviation (SD). All analyses were performed using SPSS (version 22.0) and R software 4.4.1 (www.r-project.org/).

## 3. Results

### 3.1. Subsection

#### 3.1.1. Basic Information

An unselected group of 170 patients at Peking University Third Hospital provided informed consent for this prospective cohort study. Finally, 162 patients finished the follow up, and 8 cases (4.7%) were lost. As of 6 September 2021, which was the cutoff date for the survival analysis, 52 patients (32.1%) had reached the endpoint. Of them, 7 patients had undergone tracheotomy and 45 patients died due to disease progression. Patients were grouped according to whether or not they reached the survival endpoint. Compared to the alive group, those who reached the endpoint had significantly lower ROADS and ALSFRS-R, and higher ΔROADS and ΔFRS at the baseline (*p* < 0.001, Table 1). In addition, consistent with the previous literature [8,10], the deceased or tracheostomy group showed older age and shorter symptom duration. No significant difference was found in gender and site of onset between the two groups. We conducted questionnaire follow-ups with patients at three time points, October 2020, February 2021, and July 2021. Ultimately, 146 patients (85.9%) completed two to four follow-up assessments. The Chinese version of ROADS is included in the Supplementary Materials.

#### 3.1.2. Prognostic Utility of Baseline ROADS and ΔROADS for ALS

Cox regression analyses were conducted to assess the relationship between baseline ROADS and ΔROADS with the survival time of ALS. As a continuous variable, ROADS demonstrated an inverse association with the risk of death in a univariate Cox regression model (HR = 0.96, 95% CI = 0.94–0.97, *p* < 0.001, Table 2). Conversely, ΔROADS emerged as a significant risk factor for ALS (HR = 1.30, 95% CI = 1.15–1.43, *p* < 0.001, Table 2). To further explore the relationship of ROADS and its rate to survival, patients were categorized into three subgroups based on ROADS and ΔROADS tertiles. Patients with the mildest disease, the 1st tertile with the ROADS value ≥ 82 and 1st tertile with the ΔROADS value < 1.48 were used as reference groups. Univariate Cox regression analyses demonstrated a progressively increasing HR of mortality from the 1st to the 3rd tertiles of both ROADS (62 ≤ ROADS < 82 vs. Reference, HR = 4.20, *p* = 0.010; ROADS < 62 vs. Reference, HR = 11.23, *p* < 0.001) and ΔROADS (1.48 ≤ ΔROADS < 3.29 vs. Reference, HR = 5.07, *p* = 0.001; ΔROADS ≥ 3.29 vs. Reference, HR = 6.46, *p* < 0.001). Regarding ALSFRS-R, it served as a positive predictor of survival when included as a continuous variable in Cox models (Table 2), aligning with previous studies [8,10]. However, when applying the same classification for the ALSFRS-R tertiles, only the 3rd tertile exhibited a significantly increased risk of death compared with the 1st tertile. No significant difference in the Cox model was observed between the highest and middle tertiles (24 ≤ ALSFRS-R < 35 vs. Reference, HR = 1.70, *p* = 0.26; ALSFRS-R < 24 vs. Reference, HR = 6.17, *p* < 0.001, Table 2). Other significant covariates included age and symptom duration. Older age and shorter symptom duration were identified as negative prognostic markers in ALS. In contrast, gender and onset were not significant predictors of survival in the present cohort.

**Figure 2:**
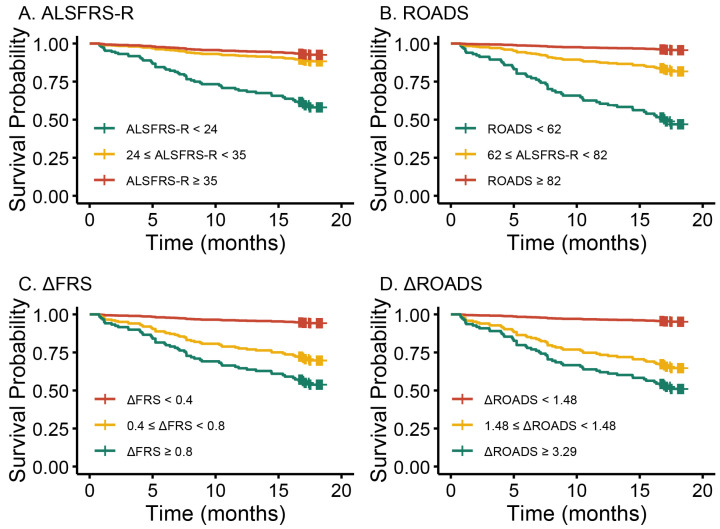
Multivariate survival curves from Cox regression analyses stratified by tertiles of ALSFRS-R (**A**), ROADS (**B**), ΔFRS (**C**), and ΔROADS (**D**).

Next, multivariate Cox regression models were employed to explore the relationship between ALS survival and above variables. The results demonstrated that, after adjusting for age, gender, site of onset, and symptom duration, ROADS was an independent favorable predictor of prognosis (HR = 0.95, 95% CI = 0.94–0.97, *p* < 0.001; Table 3) and ΔROADS was an independent adverse predictor of survival (HR = 1.26, 95% CI = 1.10–1.45, *p* < 0.001; Table 3). Furthermore, Cox regression by tertile revealed that the risk of death increased as ROADS decreased and ΔROADS increased. Importantly, consistent with results from univariate Cox analyses, for ALSFRS-R, only the 3rd tertile group exhibited a significantly higher risk of mortality, whereas the 2nd tertile group did not (24 ≤ ALSFRS-R < 35 vs. Reference, HR = 1.63, *p* = 0.32; ALSFRS-R < 24 vs. Reference, HR = 7.73, *p* < 0.001, Table 3). Multivariate survival curves generated from Cox regression analyses were shown in Figure 2A–D. They demonstrated a significant difference in survival probabilities among the three subgroups, which were categorized based on either ROADS or ΔROADS (log-rank test, *p* < 0.001, Figure 2B,D). As shown in Figure 2, ΔROADS exhibits a similar pattern to ΔFRS. The survival curves of the 1st and 2nd tertile groups of ROADS were distinguishable (Figure 2B), while as for ALSFRS-R, the survival curves of the 1st and 2nd tertile groups were relatively close (Figure 2A). These imply that both ROADS and ΔROADS are independent predictors of ALS prognosis. A higher ROADS and a lower ΔROADS at baseline are associated with a better prognosis. Their predictive performance is comparable to ALSFRS-R and ΔFRS. Further, in patients with higher scores, indicating milder disease, ROADS may potentially be more sensitive than ALSFRS-R in predicting survival.

#### 3.1.3. Longitudinal Analyses of ROADS to Measure Disease Progression

**Figure 3:**
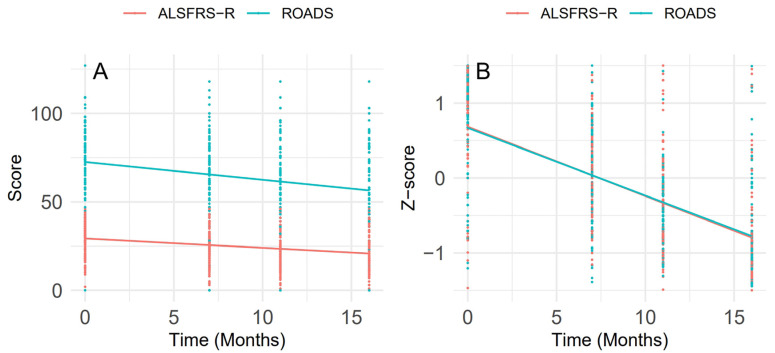
Longitudinal ALSFRS-R and ROADS changes over time. Estimates from Linear mixed-effects models of the longitudinal changes in measurements. (**A**) Mean ALSFRS-R and ROADS over time. (**B**) Mean z-score of ALSFRS-R and ROADS over time.

In this cohort, 146 patients completed longitudinal follow-up assessments during the 18-month period. Both ROADS and ALSFRS-R scores decreased over time (Figure 3A). The global slopes for monthly change were −0.68 for ALSFRS-R and −1.16 for ROADS. This means that ALSFRS-R decreased by 0.68 points per month and ROADS decreased by 1.16 points per month. The fixed effects for time of both scores were significant (*p* < 0.001), indicating a significant decline over time. The rate of change was faster in ROADS than ALSFRS-R (*p* < 0.001). However, the difference was due to the overall higher score of ROADS compared to ALSFRS-R. After Z-score standardization, the slopes for both ROADS and ALSFRS-R became nearly identical: −0.091 for ALSFRS-R and −0.088 for ROADS. There was no significant difference between them (Figure 3B). These results suggest that ROADS has the ability to capture disease progression over time, which is similar to ALSFRS-R.

## 4. Discussion

Our investigation explores the role of Chinese ROADS in assessing ALS disease progression and the relationship between ROADS and survival. Notably, this is the first to assess the correlation of the ROADS and ALS survival. While traditional measures such as ALSFRS-R have been foundational [12], they exhibit limitations, including multidimensionality and insensitivity to certain nuances of progression [11,12,13]. In this study, ROADS emerges as a promising alternative measure, rooted in Rasch analysis principles [17].

Our study defines ΔROADS for the first time. The results verify that both ROADS and ΔROADS are prognostic markers in ALS, demonstrating a level of comparability with ALSFRS-R and ΔFRS. The findings indicate that higher ROADS and lower ΔROADS at baseline are associated with longer survival in a Chinese ALS clinic population. The ability to predict survival outcomes is a critical aspect of any ALS assessment tool, and the correlation observed between ROADS and ΔROADS and survival time adds to its credibility in capturing disease progression. It is worth noting that, when divided into subgroups according to the ALSFRS-R tertiles, survival curves show no difference in survival probability between the highest score subgroup and the middle score subgroup. As for ROADS, survival curves show a significant difference among the three subgroups. Compared with the ROADS, the HRs of ALSFRS-R tertiles were relatively in close proximity, thus the distinction among the three groups may be less obvious than the ROADS tertiles. This suggests that, compared with ALSFRS-R, ROADS may be more sensitive as a prognostic indicator in ALS patients with mild disease. Several factors may contribute to this finding. For instance, the method used to develop ROADS ensures a broader range of item targeting than ALSFRS-R, which may enable it to better distinguish between levels of overall disability among respondents [17,23]. Additionally, the 28-questioned ROADS has more items than the ALSFRS-R questionnaire, which could mean that multiple items in ROADS evaluate the same aspect of disease dysfunction. Therefore, the use of ROADS may provide more detailed information regarding disease dysfunction, increasing the likelihood of detecting changes when they occur.

Moreover, we examined the longitudinal performance of Chinese ROADS in the follow-up period. The results demonstrate its robust performance in measuring disease progression, comparable to ALSFRS-R. Despite several previous studies validating ROADS’ responsiveness to change over time [26,27,28], the Chinese ROADS, a translated and culturally adapted scale, lacked prior longitudinal validation in the Chinese population. The stability across diverse populations and cultural contexts reinforces its reliability in longitudinally monitoring ALS disability.

ALS presently lacks sensitive and specific biomarkers due to its strong biological and clinical heterogeneity [30,31,32]. Hence, selection of an appropriate quantitate measure is vital for monitoring disease progression in clinic and capturing potential drug therapeutic efficacy. As a key outcome measure, problems of multidimensionality [11,12], non-linearity [13], and floor and ceiling effect [33,34,35] have challenged the efficacy of ALSFRS-R in trials. These limitations may dilute possible treatment effects and result in failures in new drug research [16,36]. The recently developed ROADS validated by Rasch analysis has been proved to have several theoretical advantages over the ALSFRS-R in previous studies [17,23]. The present longitudinal study demonstrates that ROADS shows a good correlation with ALS survival. And, it has the potential to provide better discrimination for predicting mortality in ALS patients with mild disease. This lays the groundwork for using the ROADS as a valuable outcome measure in future clinical applications and ALS trials. On the other hand, compared to participants in clinical trials who must meet restrictive inclusion criteria, our sample from a real-world clinical setting is probably more representative of the general ALS population.

There are several limitations in our study. A relatively small sample size, a short follow-up, and few events may limit statistical power. Despite this, we also detected a significant prognostic value of the ROADS. In addition, we had other limitations such as a lack of information on important confounders, such as forced vital capacity, treatment information, and weight loss. Therefore, our result is only observed in this single-center study with a small sample size. In addition, this study was conducted in a Chinese cohort. Our finding requires further verification with more detailed clinical information in larger and more heterogenous data sets including diverse patients with other races and ethnicities. In future research, we plan to expand the sample size and collect more information to confirm the performance of ROADS.

## 5. Conclusions

In conclusion, our study has validated the significance of ROADS in assessing ALS progression and predicting prognosis, demonstrating its comparable performance with ALSFRS-R. Therefore, ROADS may serve as a valuable measurement tool to monitor disease progression in clinical trials and clinical settings, potentially complementing ALSFRS-R. This study was conducted in Chinese patients. The efficacy of ROADS in diverse populations needs to be further validated. And its performance in assessing therapeutic efficacy in ALS needs to be evaluated in future clinical trials.

## Figures and Tables

**Table 1:** Baseline demographic and clinical characteristics of study participants (*n* = 162).

Variable	Total	Deceased or Tracheostomy	Alive Without Tracheostomy
No. of patients, n (%)	162	52 (32.1)	110 (67.9)
Baseline age *, y, mean (SD)	55.0 (12.2)	59.9 (9.9)	52.7 (12.6)
Male, n (%)	102 (63.0)	34 (65.4)	68 (61.8)
Site of onset, n (%)			
Spinal cord	134 (82.7)	40 (76.9)	94 (85.5)
Bulbar	28 (17.3)	12 (23.1)	16 (14.5)
Symptom duration ^†^, m, mean (SD)	44.5 (40.3)	31.4 (20.9)	50.6 (45.6)
Baseline normed ROADS ^†^, mean (SD)	70.9 (20.3)	57.7 (18.6)	77.1 (18.0)
Baseline ALSFRS-R ^†^, mean (SD)	27.9 (9.8)	21.9 (9.6)	30.8 (8.6)
Baseline ΔROADS ^†^, mean (SD)	2.72 (2.00)	3.68 (2.24)	2.26 (1.70)
Baseline FRS ^†^, mean (SD)	0.76 (0.65)	1.15 (0.82)	0.57 (0.45)
Survival, m, mean (SD)	—	7.6 (5.0)	17.6 (0.59)

Abbreviation: ALSFRS-R, revised Amyotrophic Lateral Sclerosis Functional Rating Scale; ROADS, Rasch-Built Overall Amyotrophic Lateral Sclerosis Disability Scale; SD, standard deviation. * *p* = 0.02, † *p* < 0.001 for the comparison of deceased or tracheostomy vs. alive without tracheostomy (Student’s *t*-test for normal data and Wilcoxon’s rank sum test for non-normal data).

**Table 2:** Univariate Cox regression analyses for death or tracheostomy (n = 162).

Variable	Hazard Ratio (95% CI)	*p* Value
Age, year		
<55	1.00 (Reference)	NA
≥55	2.55 (1.43–4.56)	0.002
Gender		
Male	1.00 (Reference)	NA
Female	0.89 (0.50–1.58)	0.70
Site of onset		
Spinal cord	1.00 (Reference)	NA
Bulbar	1.45 (0.76–2.77)	0.27
Symptom duration	0.99 (0.98–1.00)	0.012
ROADS	0.96 (0.94–0.97)	<0.001
ALSFRS-R	0.92 (0.89–0.94)	<0.001
ROADS tertiles		
≥82	1.00 (Reference)	NA
≥62, <82	4.20 (1.41–12.51)	0.010
<62	11.23 (3.95–31.92)	<0.001
ALSFRS-R tertiles		
≥35	1.00 (Reference)	NA
≥24, <35	1.70 (0.67–4.33)	0.26
<24	6.17 (2.72–13.98)	<0.001
ΔROADS	1.30 (1.15–1.43)	<0.001
ΔFRS	2.68 (1.94–3.71)	<0.001
ΔROADS tertiles		
<1.48	1.00 (Reference)	NA
≥1.48, <3.29	5.07 (1.91–13.46)	0.001
≥3.29	6.46 (2.48–16.83)	<0.001
ΔFRS tertiles		
<0.40	1.00 (Reference)	NA
≥0.40, <0.80	4.32 (1.61–11.60)	0.004
≥0.80	7.33 (2.83–19.01)	<0.001

Abbreviation: ALSFRS-R, revised Amyotrophic Lateral Sclerosis Functional Rating Scale; ROADS: Rasch-Built Overall Amyotrophic Lateral Sclerosis Disability Scale; CI, confidence interval.

**Table 3:** Multivariate Cox regression analyses for death or tracheostomy (n = 162).

Variable	Hazard Ratio (95% CI)	*p* Value
ROADS	0.95 (0.94–0.97)	<0.001
ALSFRS-R	0.90 (0.87–0.93)	<0.001
ROADS tertiles		<0.001
≥82	1.00 (reference)	NA
≥62, <82	4.82 (1.60–14.54)	0.005
<62	17.99 (6.17–52.47)	<0.001
ALSFRS-R tertiles		<0.001
≥35	1.00 (reference)	NA
≥24, <35	1.63 (0.63–4.20)	0.32
<24	7.73 (3.34–17.94)	<0.001
ΔROADS	1.26 (1.10–1.45)	0.001
ΔFRS	2.54 (1.73–3.73)	<0.001
ΔROADS tertiles		
<1.48	1.00 (Reference)	NA
≥1.48, <3.29	8.71 (2.38–31.10)	0.001
≥3.29	13.90 (3.36–57.55)	<0.001
ΔFRS tertiles		
<0.40	1.00 (Reference)	NA
≥0.40, <0.80	5.90 (1.86–18.69)	0.003
≥0.80	10.90 (3.10–38.73)	<0.001

Abbreviation: ALSFRS-R, revised Amyotrophic Lateral Sclerosis Functional Rating Scale; ROADS, Rasch-Built Overall Amyotrophic Lateral Sclerosis Disability Scale; HR, hazard ratio; CI, confidence interval.

## Data Availability

The raw data supporting the conclusions of this article will be made available by the authors on request.

## Supplementary Materials

The following supporting information can be downloaded at: https://www.mdpi.com/article/10.3390/biomedicines13010178/s1.
