# Supplementary material for: Rasch-Built Overall Amyotrophic Lateral Sclerosis Disability Scale as a Novel Tool to Measure Disease Progression
Source: Biomedicines. 2025 Jan 13;13(1):178. doi: 10.3390/biomedicines13010178 (PMC11759773; doi:10.3390/biomedicines13010178)
Supplement: Supplementary file 1 [file biomedicines-13-00178-s001.zip › ROADS量表中文版(新版).pdf]

Rasch 肌萎缩侧索硬化整体功能障碍评价量表(ROADS)

建档号：______________　　　　填表日期: _______________________

姓名：________________　　　　性别：_____出生日期：___________

简介：　这是一份关于您日常活动与健康之间关系的问卷。您的回答有助于医生了解肌萎缩侧索硬化症如何影响您的日常活动，以及您能够完成日常活动的程度。

请在正确的方框内标记("√")来回答每个问题。如果您不确定完成某项活动的能力，那么您需要预估一下自己完成类似活动的能力，并选择一个最接近的答案。您需要完成所有的问题，每个问题只能选择一个答案。如果您完成某项活动的情况有波动，那么请选择通常情况下完成这项活动的能力。

请按照以下标准作答：

[2]正常：能够轻松完成——如果您完成某项活动的能力与出现肌萎缩侧索硬化症的症状之前一样，请选择此项。这表示您现在完成某项活动与发病前没有任何不同。

[1]异常：能够完成，但有难度——如果您仍可以完成某项活动，但与出现肌萎缩侧索硬化症的症状之前相比，完成起来更困难或需要花费更多的时间和精力才能完成，请选择此选项。如果您需要辅助工具或他人的帮助才能完成该项活动，也请选择此选项。

[0]无法完成——如果无法完成某项活动，请选择此选项。

项目	无法完成	异常：能够完成，但有难度	正常：能够轻松完成
1 点头、摇头？	[ ]	[ ]	[ ]
2 坐车？ 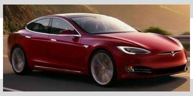	[ ]	[ ]	[ ]
3 吃馄饨？	[ ]	[ ]	[ ]
4 用勺子喝汤？	[ ]	[ ]	[ ]
5 坐在马桶上？	[ ]	[ ]	[ ]
6 吞咽药片？	[ ]	[ ]	[ ]
7 吹灭蜡烛？	[ ]	[ ]	[ ]
8 吃干的食物（如馒头、面包）？	[ ]	[ ]	[ ]
9 讲电话？	[ ]	[ ]	[ ]
10 喝完一杯水？	[ ]	[ ]	[ ]
11 饱餐一顿？	[ ]	[ ]	[ ]
12 在纸上签名？	[ ]	[ ]	[ ]
13 上床？	[ ]	[ ]	[ ]
14 在床上翻身？	[ ]	[ ]	[ ]
15 洗澡？	[ ]	[ ]	[ ]
16 使用筷子？	[ ]	[ ]	[ ]
17 在家里四处走走？	[ ]	[ ]	[ ]
18 移动一把椅子？	[ ]	[ ]	[ ]
19 在嘈杂的房间里讲话？	[ ]	[ ]	[ ]
20 连续讲话几个小时？	[ ]	[ ]	[ ]
21 剪指甲？	[ ]	[ ]	[ ]
22 爬一层楼？	[ ]	[ ]	[ ]
23 爬山坡？	[ ]	[ ]	[ ]
24 爬家用梯子？ 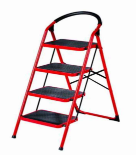	[ ]	[ ]	[ ]
25 从地上站起来？	[ ]	[ ]	[ ]
26 搬东西下楼？	[ ]	[ ]	[ ]
27 站立几个小时？	[ ]	[ ]	[ ]
28 从高架子上取下重物？	[ ]	[ ]	[ ]

原始总分__________　　　　标准总分__________

线性加权的标准 **ROADS** 得分

**ROADS** 原始总分	**ROADS Logit** 值	标准总分	**ROADS** 原始总分	**ROADS Logit** 值	标准总分
0	-6.15	0	29	0.15	73
1	-4.91	14	30	0.25	74
2	-4.16	23	31	0.35	75
3	-3.69	28	32	0.45	76
4	-3.35	32	33	0.55	78
5	-3.06	36	34	0.65	79
6	-2.82	39	35	0.75	80
7	-2.6	41	36	0.85	81
8	-2.41	43	37	0.95	82
9	-2.23	45	38	1.06	83
10	-2.07	47	39	1.17	85
11	-1.91	49	40	1.28	86
12	-1.76	51	41	1.39	87
13	-1.63	52	42	1.51	89
14	-1.49	54	43	1.63	90
15	-1.36	55	44	1.75	91
16	-1.24	57	45	1.89	93
17	-1.12	58	46	2.03	95
18	-1.01	60	47	2.18	96
19	-0.89	61	48	2.35	98
20	-0.78	62	49	2.53	100
21	-0.67	63	50	2.73	103
22	-0.57	65	51	2.96	105
23	-0.46	66	52	3.23	109
24	-0.36	67	53	3.57	113
25	-0.25	68	54	4.04	118
26	-0.15	69	55	4.79	127
27	-0.05	71	56	6.04	141
28	0.05	72			
