# Supplementary material for: Rasch-Built Overall Amyotrophic Lateral Sclerosis Disability Scale as a Novel Tool to Measure Disease Progression
Source: Biomedicines. 2025 Jan 13;13(1):178. doi: 10.3390/biomedicines13010178 (PMC11759773; doi:10.3390/biomedicines13010178)
Supplement: Supplementary file 1 [file biomedicines-13-00178-s001.zip › Chinese version of ROADS.pdf]

Chinese Rasch Overall ALS Disability Scale (ROADS)

MRN: ______________________________ **TODAY'S DATE:** ____________________

NAME: _____________________________ **DATE OF BIRTH:**_____________________

INSTRUCTIONS: This is a questionnaire about the relationship between your daily activities and your health. Your answers give information about how your ALS affects your daily and social activities and to what degree you are able to perform your usual activities.

Answer each question by marking the correct box ("X"). If you are not sure about your ability to perform a task, you are still requested to mark an answer that comes as close as possible to your judged ability to complete such a task. All questions should be completed. You can only choose one answer for each question. If your situation fluctuates, your answer should be based on how you *usually* perform the task.

Please use the following guidelines for selecting your answer choice:

[2] <u>Normal: able to perform without difficulty</u>- select this option if you perform the task as easily and as quickly as you did before you had ALS symptoms. This option indicates that you perform the task no differently now then you did before you had ALS symptoms.

[1] <u>Abnormal: able to perform, but with difficulty</u>- select this option if you can still perform the task, but it is harder to perform now or takes more time or effort compared to before you had ALS symptoms. Also select this option if you perform a task with the assistance of a device or with the assistance of another person.

[0] <u>Unable to perform</u>- select this option if you cannot perform the task.

Task		Unable to perform [0]	Abnormal: able to perform but with difficulty [1]	Normal: able to perform without difficulty [2]
1	nod yes or no?	[ ]	[ ]	[ ]
2	ride in a car?	[ ]	[ ]	[ ]
3	eat wonton?	[ ]	[ ]	[ ]
4	drink soup with a spoon?	[ ]	[ ]	[ ]
5	sit on a toilet?	[ ]	[ ]	[ ]
6	swallow pills?	[ ]	[ ]	[ ]
7	blow out a candle?	[ ]	[ ]	[ ]
8	eat dry food (e.g. steamed bun, bread)?	[ ]	[ ]	[ ]
9	speak on the phone?	[ ]	[ ]	[ ]
10	drink out of a glass?	[ ]	[ ]	[ ]
11	eat a large meal?	[ ]	[ ]	[ ]
12	sign name on paper?	[ ]	[ ]	[ ]
13	get into bed?	[ ]	[ ]	[ ]
14	roll over in bed?	[ ]	[ ]	[ ]
15	take a shower?	[ ]	[ ]	[ ]
16	use chopsticks?	[ ]	[ ]	[ ]
17	walk around your home?	[ ]	[ ]	[ ]
18	move a chair?	[ ]	[ ]	[ ]
19	speak in a loud room?	[ ]	[ ]	[ ]
20	speak for hours?	[ ]	[ ]	[ ]
21	clip nails?	[ ]	[ ]	[ ]
22	walk up 1 flight of stairs?	[ ]	[ ]	[ ]
23	walk up a hill?	[ ]	[ ]	[ ]
24	climb a stepstool?	[ ]	[ ]	[ ]
25	get up from the floor?	[ ]	[ ]	[ ]
26	carry an object going downstairs?	[ ]	[ ]	[ ]
27	stand for hours?	[ ]	[ ]	[ ]
28	get heavy objects off a high shelf?	[ ]	[ ]	[ ]

RAW TOTAL ___________ NORMED TOTAL___________

Linearly-weighted normed ROADS scores

ROADS raw sum-score	Rasch logit measure	Normed whole number score
0	-6.15	0
1	-4.91	14
2	-4.16	23
3	-3.69	28
4	-3.35	32
5	-3.06	36
6	-2.82	39
7	-2.6	41
8	-2.41	43
9	-2.23	45
10	-2.07	47
11	-1.91	49
12	-1.76	51
13	-1.63	52
14	-1.49	54
15	-1.36	55
16	-1.24	57
17	-1.12	58
18	-1.01	60
19	-0.89	61
20	-0.78	62
21	-0.67	63
22	-0.57	65
23	-0.46	66
24	-0.36	67
25	-0.25	68
26	-0.15	69
27	-0.05	71
28	0.05	72

ROADS raw sum-score	Rasch logit measure	Normed whole number score
29	0.15	73
30	0.25	74
31	0.35	75
32	0.45	76
33	0.55	78
34	0.65	79
35	0.75	80
36	0.85	81
37	0.95	82
38	1.06	83
39	1.17	85
40	1.28	86
41	1.39	87
42	1.51	89
43	1.63	90
44	1.75	91
45	1.89	93
46	2.03	95
47	2.18	96
48	2.35	98
49	2.53	100
50	2.73	103
51	2.96	105
52	3.23	109
53	3.57	113
54	4.04	118
55	4.79	127
56	6.04	141
